# Targeted control of supporting pathways in paclitaxel biosynthesis with CRISPR-guided methylation

**DOI:** 10.3389/fbioe.2023.1272811

**Published:** 2023-10-17

**Authors:** Cassandra Brzycki Newton, Eric M. Young, Susan C. Roberts

**Affiliations:** Department of Chemical Engineering, Worcester Polytechnic Institute, Worcester, MA, United States

**Keywords:** plant cell culture, secondary metabolism, metabolic engineering, paclitaxel, CRISPR

## Abstract

**Introduction:** Plant cell culture biomanufacturing is rapidly becoming an effective strategy for production of high-value plant natural products, such as therapeutic proteins and small molecules, vaccine adjuvants, and nutraceuticals. Many of these plant natural products are synthesized from diverse molecular building blocks sourced from different metabolic pathways. Even so, engineering approaches for increasing plant natural product biosynthesis have typically focused on the core biosynthetic pathway rather than the supporting pathways.

**Methods:** Here, we use both CRISPR-guided DNA methylation and chemical inhibitors to control flux through the phenylpropanoid pathway in *Taxus chinensis*, which contributes a phenylalanine derivative to the biosynthesis of paclitaxel (Taxol), a potent anticancer drug. To inhibit PAL, the first committed step in phenylpropanoid biosynthesis, we knocked down expression of PAL in *Taxus chinensis* plant cell cultures using a CRISPR-guided plant DNA methyltransferase (NtDRM). For chemical inhibition of downstream steps in the pathway, we treated *Taxus chinensis* plant cell cultures with piperonylic acid and caffeic acid, which inhibit the second and third committed steps in phenylpropanoid biosynthesis: cinnamate 4-hydroxylase (C4H) and 4-coumaroyl-CoA ligase (4CL), respectively.

**Results:** Knockdown of PAL through CRISPR-guided DNA methylation resulted in a profound 25-fold increase in paclitaxel accumulation. Further, through the synergistic action of both chemical inhibitors and precursor feeding of exogenous phenylalanine, we achieve a 3.5-fold increase in paclitaxel biosynthesis and a similar reduction in production of total flavonoids and phenolics. We also observed perturbations to both activity and expression of PAL, illustrating the complex transcriptional co-regulation of these first three pathway steps.

**Discussion:** These results highlight the importance of controlling the metabolic flux of supporting pathways in natural product biosynthesis and pioneers CRISPR-guided methylation as an effective method for metabolic engineering in plant cell cultures. Ultimately, this work demonstrates a powerful method for rewiring plant cell culture systems into next-generation chassis for production of societally valuable compounds.

## 1 Introduction

Plants produce an incredible variety of unique compounds with bioactive properties that have many industrial uses as pesticides, food additives, cosmetics, and pharmaceuticals (Zhu et al., 2021). However, sustainable industrial production of these plant natural products is often challenging. Complex stereochemistry limits yields from chemical synthesis, while lengthy or uncharacterized biosynthetic pathways make it difficult or impossible to produce certain plant natural products heterologously (Zhang et al., 2022). Plant cell culture (PCC) is therefore becoming an attractive option for sustainable biomanufacturing of complex and high-value plant secondary metabolites, including small molecule therapeutics, vaccine adjuvants, and nutraceuticals. One key challenge with optimizing plant cell systems for production of a single metabolite is that plants often have a multitude of different cooperating and competing secondary metabolic pathways whose interactions must be considered in the context of metabolic engineering. However, these complex interactions are often overlooked in favor of solely focusing on manipulating the pathway of interest. To enable more complete understanding and control of secondary metabolism and achieve the highest possible yields of products of interest, approaches for manipulating cooperating and competing metabolic pathways are needed.

One example of a PCC chassis where manipulating cooperating and competing pathways is vital is *Taxus chinensis,* which along with other species in the *Taxus* genus produces a chemotherapeutic drug called paclitaxel as a natural product (Wilson and Roberts, 2012). Production of this drug using *Taxus* PCC is one of the most wildly successful commercializations of PCC technology to date, with the world’s largest supplier of paclitaxel (Phyton Biotech) using a PCC process (Wilson and Roberts, 2012). The biosynthetic pathway for synthesis of paclitaxel is highly complex—it is a cyclic diterpenoid that is synthesized from the terpene precursors IPP and DMAPP via a branching pathway that includes 19 putative enzymatic steps, 15 of which have been characterized (Sanchez-Muñoz et al., 2020). While much effort has been devoted to studying paclitaxel biosynthesis, comparatively little work has been done to understand how paclitaxel biosynthesis interacts with other secondary metabolic pathways that may compete for cellular resources (McKee et al., 2021). Thus, to improve yields of paclitaxel from PCC, it is vital to understand and manipulate not only paclitaxel biosynthesis, but also the many other metabolic pathways that make up primary and secondary metabolism.

Paclitaxel biosynthesis is hypothesized to interact with phenylpropanoid biosynthesis, as both pathways utilize the aromatic amino acid phenylalanine as a common precursor (McKee et al., 2021). In paclitaxel biosynthesis, α-phenylalanine is converted to β-phenylalanine via the enzyme phenylalanine aminomutase (PAM), which is then ligated to acetyl-CoA and attached to the taxane backbone via the enzyme BAPT (Sanchez-Muñoz et al., 2020). Phenylalanine is also a key building block used in synthesis of thousands of different phenylpropanoid compounds, including flavonoids and phenolics, which play roles as pigments, signaling molecules, and antioxidants, as well as lignin, a major component of the plant cell wall (Liu et al., 2021). The first committed step in synthesis of all phenylpropanoid-derived compounds is the conversion of α-phenylalanine to cinnamic acid by the enzyme phenylalanine ammonia-lyase (PAL) (Liu et al., 2021; McKee et al., 2021), which is known to play a vital role in controlling the division of carbon flux from primary to secondary metabolism in many plant species (Kim and Hwang, 2014; Feduraev et al., 2020; Liu et al., 2021). PAL is an especially critical enzyme because of its important role in controlling flux to lignin biosynthesis, which affects plant cell wall architecture and susceptibility to infestations and infections (Pant et al., 2021; Pant and Huang, 2022). Cinnamic acid is then converted to coumaric acid via the enzyme cinnamate 4-hydroxylase (C4H), which is then converted to coumaroyl-CoA via the enzyme 4-coumaroyl-CoA ligase (4CL) (Li et al., 2015; Liu et al., 2021; McKee et al., 2021). Cinnamic acid, coumaric acid, and coumaroyl-CoA all serve as precursors that can ultimately feed into phenylpropanoid biosynthesis through complex, branching pathways (Liu et al., 2021). Due to the involvement of the phenylpropanoid pathway in producing lignin, in many plants there is a substantial amount of carbon flux that is routed to producing phenylalanine (Yoo et al., 2013). Therefore, rerouting even a small fraction of this phenylalanine away from phenylpropanoid metabolism and toward paclitaxel biosynthesis could have the potential to significantly improve paclitaxel yields.

It is well-established that elicitation of *Taxus* spp. PCCs with methyl jasmonate or other elicitors causes general upregulation of secondary metabolism, including both taxane biosynthesis and phenylpropanoid biosynthesis (Bulgakov et al., 2011; Li et al., 2012; Zhou et al., 2019). While these studies indicate that these pathways have some degree of co-regulation, the exact effect of targeted inhibition of phenylpropanoid metabolism on taxane biosynthesis remains inconclusive. One previous study in *Taxus* found that inhibition of phenylpropanoid biosynthesis with either cinnamic acid or α-aminooxyacetic acid (AOA) significantly decreased paclitaxel biosynthesis (Brincat et al., 2002), while another study suggests no significant relationship between PAL activity and taxane biosynthesis (Bamneshin et al., 2022). Despite these results, patents on *Taxus* plant cell culture technology report that inhibitors of phenylpropanoid metabolism (in particular, cinnamic acid derivatives) can be used to increase paclitaxel production (Bringi et al., 2012). To further understand the relationship amongst these cooperating pathways, we studied the effect of inhibition of three different steps in early-stage phenylpropanoid biosynthesis (PAL, C4H, and 4CL) on taxane metabolism.

Pathway inhibition can be accomplished using two general classes of methods: genetic methods that manipulate gene expression and non-genetic methods that manipulate enzyme activity through chemical inhibition. Manipulation of metabolism using non-genetic methods can be advantageous for non-model organisms because these methods can be used to inhibit enzymes for which the exact gene sequence is not known for a particular species. While the gene for PAL has been identified in multiple *Taxus* species (Dong et al., 2016; Bamneshin et al., 2022)*,* genes encoding the following two downstream steps in phenylpropanoid biosynthesis (C4H and 4CL) have yet to be discovered, necessitating this approach. As most current research on phenylpropanoid biosynthesis in PCCs has been centered on chemical inhibition of PAL (Brincat et al., 2002; Su et al., 2015; Andi et al., 2019; Bamneshin et al., 2022), we instead studied the effects of inhibiting the downstream enzymes C4H and 4CL. Piperonylic acid is one of the most potent irreversible inhibitors of C4H and has been used effectively to inhibit phenylpropanoid biosynthesis in BY-2 tobacco cell suspensions (Schalk et al., 1998). Caffeic acid is an alternative substrate of 4CL (*11*) that has been shown to decrease 4CL enzymatic activity through competitive inhibition (Chen et al., 2013) and has been used to manipulate flux to lignin biosynthesis in soybean plants (Bubna et al., 2011). Neither of these inhibitors have been studied to date in *Taxus* PCC.

In addition to chemical inhibition, we also sought to manipulate the phenylpropanoid pathway using a genetic approach. Two of the most widely used methods for inhibiting gene expression are RNA interference (RNAi) and CRISPR interference (CRISPRi) (Brzycki et al., 2021). RNAi causes degradation of mRNA transcripts, thus preventing translation (Fire et al., 1998), while CRISPRi prevents transcription of a target sequence through steric hindrance (Larson et al., 2013). A third, emerging approach involves controlling gene expression epigenetically through linking catalytically dead Cas9 (dCas9) proteins to catalytic domains that add or remove epigenetic markers, such as DNA methylation and histone acetylation (Brzycki et al., 2021). For example, these effector domains could include DRM methyltransferases, which methylate DNA (Papikian et al., 2019), TET family enzymes, which demethylate DNA (Gallego-Bartolomé et al., 2018), and p300 histone acyltransferases, which participate in histone acetylation (Hilton et al., 2015). Similar to CRISPRi, these approaches regulate gene expression on the transcriptional level, while RNAi regulates gene expression on the translational level. However, CRISPR-based epigenetic engineering has the additional advantage of being able to directly manipulate specific epigenetic markers that are known to play roles in regulation of biological processes, such as cell aging and differentiation (Brzycki et al., 2021).

While CRISPR-mediated epigenetic engineering has been used effectively to control gene expression in the model plant *Arapidopsis thaliana* (Papikian et al., 2019), it has not yet been explored as a tool for metabolic engineering in plant cell culture (Perez-Matas et al., 2023). This approach could be particularly advantageous for engineering PCCs because methylation has been established as a key regulator of gene expression that increases as plant cell lines are continuously subcultured and removing DNA methylation has been associated with rescuing gene expression in epigenetically silenced plant cell lines (Fu et al., 2012; Tyunin et al., 2012). Therefore, developing approaches for specifically manipulating DNA methylation rather than transcription more generally may be advantageous for PCC systems. To this end, we sought to explore a novel technique for manipulating phenylpropanoid metabolism through CRISPR-mediated targeted DNA methylation of PAL, the first committed step in phenylpropanoid biosynthesis and the only phenylpropanoid pathway enzyme currently characterized in *Taxus chinensis* (Dong et al., 2016). Through studying the effects of inhibiting these first three steps in phenylpropanoid biosynthesis, we aim to gain a more complete understanding of the relationship between phenylpropanoid and taxane metabolism than through manipulating PAL alone.

Here, we explore genetic and non-genetic inhibition of the first three committed steps in phenylpropanoid biosynthesis (PAL, C4H, and 4CL) to elucidate its co-regulation with taxane biosynthesis. Using chemical inhibitors and precursor feeding with phenylalanine, we find that we can achieve up to a 3.5-fold increase in paclitaxel accumulation. The increase in paclitaxel production observed with chemical inhibition is amplified when cultures are fed with exogenous phenylalanine, indicating that phenylalanine availability likely plays a regulatory role in directing flux between phenylpropanoid and taxane biosynthesis. Unexpectedly, we also found that accumulation of the taxane pathway intermediates 10-deacetylbaccatin III and baccatin III also increased significantly when phenylpropanoid metabolism was disrupted, despite these steps being upstream of where the phenylalanine side chain attaches to the taxane backbone. We propose that this differential accumulation is due to the involvement of phenylpropanoid metabolism in biosynthesis of plant hormones that regulate the stress response, specifically salicylic acid. We also show that CRISPR-mediated DNA methylation upstream of the PAL gene disrupts phenylpropanoid metabolism and consequently increases production of paclitaxel by up to 25-fold. This provides a proof-of-concept for the application of CRISPR tools to PCCs and demonstrates that targeted repression of pathways can be a powerful tool for manipulating metabolism. These results provide insights on the complex interactions between phenylpropanoid biosynthesis and paclitaxel biosynthesis in *Taxus* species and more generally demonstrate the efficacy of several novel genetic and non-genetic approaches for manipulating flux between competing metabolic pathways in PCC.

## 2 Materials and methods

### 2.1 Plant cell line initiation and maintenance

*Taxus chinensis* cell line 48.82A.3s was initiated from tree 48–82*A at the Arnold Arboretum of Harvard University (Cambridge, MA). Healthy light green needle samples were surface-sterilized by incubating for 10 min at room temperature in a 10% bleach solution and rinsed 3 times with sterile water. Needles were cut lengthwise with a sterile scalpel and plated on solid callus initiation media containing 20 g/L sucrose (Caisson Labs), 4 g/L Gelzan (Phytotechnology Laboratories), 3.21 g/L Gamborg B5 Basal Medium (Caisson Labs), 0.5 g/L casamino acids (Fisher BioReagents), 2.4 mg/L picloram (TCI America), and 2 μg/L 6-benzylaminopurine (Sigma Life Sciences), pH 5.5. Plates were incubated at 23°C in the dark for approximately 4–6 weeks, or until the formation of friable callus. Callus tissue was gently separated from the needles using a sterile spatula and transferred to freshly prepared callus initiation media every 4 weeks until sufficient biomass was obtained to transfer into suspension cultures. Approximately 3 g of wet biomass was inoculated into 50 mL of *Taxus* growth medium (20 g/L sucrose, 0.5 mg/L naphthaleneacetic acid, 22.5 μg/L 6-benzylaminopurine, 3.21 g/L Gamborg B5 Basal Medium, pH 5.5) supplemented with a ×20 sterile-filtered antioxidant solution (2.5 g/L ascorbic acid, 2.5 g/L citric acid, 14.6 g/L L-glutamine) at 5% v/v. Cultures were transferred to an incubator-shaker at 23°C and 125 rpm and maintained in suspension via biweekly subculturing as previously described (Kolewe et al., 2011).

### 2.2 Phenylalanine precursor feeding and inhibitor experiments

For experiments with phenylalanine, piperonylic acid, and caffeic acid, ×20 antioxidant solutions were prepared as described in Section 2.1 with the addition of 20 mM phenylalanine, 2 mM piperonylic acid (PA), and/or 2 mM caffeic acid (CA). At the beginning of a new subculture cycle, *Taxus chinensis* 48.82A.3s cultures were subcultured into growth media supplemented with these antioxidants and incubated at 23°C and 125 rpm for 21 days. 1 mL well-mixed culture samples were then taken for measurement of taxanes, cinnamic acid, and coumaric acid via UPLC, phenolic and flavonoid assays, gene expression via RT-qPCR, and PAL activity. Samples for UPLC and phenolic and flavonoid assays were dried overnight in an evaporative centrifuge and then frozen at −80°C. Samples for gene expression were allowed to sit at room temperature for several minutes, or until cells settled to the bottom of the microcentrifuge tube. The supernatant was removed using a pipette and samples were then immediately frozen at −80°C. Samples for the PAL activity assay were processed immediately (see Section 2.5).

### 2.3 Quantification of taxanes, coumaric acid, and cinnamic acid

Taxanes (including paclitaxel, baccatin III, 10-deacetyltaxol, cephalomannine, and 10-deacetylbaccatin III) were extracted from 1 mL of well-mixed culture and quantified using a previously described method (Naill and Roberts, 2004). Dried cell samples stored at −80°C were resuspended in 1 mL acidified methanol (0.01% acetic acid in methanol), broken up with a metal spatula, then sonicated three times for 20 min per cycle in a sonication bath. When the pellets were evenly and sufficiently pulverized to homogenous sand-like granules, the non-dissolved cell matter was separated from the methanol extract by centrifugation at 20,000 ×g for 10 min. This extract was evaporated using an evaporative centrifuge and samples were then resuspended in 100 μL 25/35/40% (v/v) methanol/acetonitrile/water. Taxanes were quantified using a Waters Acquity UPLC H-Class system equipped with a 1.7 μm Acquity UPLC BEH C18 column. Separation was performed using a 10 μL injection with a gradient of 30%–80% acetonitrile in water over 4 min at a flow rate of 0.35 mL/min. Peak areas at 228 nm were used to determine the concentration of each taxane.

For cinnamic acid and coumaric acid, the same samples were run using a gradient HPLC method utilizing 0.5% phosphoric acid in water (mobile phase A) and acetonitrile (mobile phase B) adapted for use in UPLC from Wan Mazlina Md et al. (2019). Separation was performed using a 1 μL injection with a gradient of 95%–20% mobile phase A over 1.97 min at a flow rate of 0.5 mL/min. Peak areas at 280 nm were used to determine concentrations of cinnamic acid and peak areas at 310 nm were used to determine concentrations of coumaric acid. All taxane, cinnamic acid, and coumaric acid peaks were identified using retention time, UV spectra, and co-chromatography with commercially available standards.

### 2.4 Total phenolic and flavonoid assays

Spectrophotometric assays for total phenolics and flavonoids were performed based on the procedure described in McKee et al. (2021) *.* Evaporated cell samples stored at −80°C were resuspended in 1 mL acidified methanol (0.01% acetic acid in methanol), broken up with a metal spatula, then sonicated three times for 20 min per cycle in a sonication bath. When the pellets were evenly and sufficiently pulverized to homogenous sand-like granules, the non-dissolved cell matter was separated from the methanol extract by centrifugation at 20,000 ×g for 10 min.

The phenolic content per sample was determined using the Folin-Ciocalteu reagent assay (Ainsworth and Gillespie, 2007). 120 μL methanol extract was combined with 200 μL Folin-Ciocalteu reagent (0.2 N) and 960 μL sodium carbonate (700 mM) in a microcentrifuge tube. After 10 min of incubation at room temperature, any precipitant was pelleted via centrifugation at 20,000 ×g for 1 min 200 μL of supernatant was transferred to a 96-well plate in triplicate, and the absorbance was read at 750 nm using the Synergy H1 Hybrid Multi-Mode Reader (BioTek). Absorbance values were converted to concentrations using gallic acid as a phenolic compound standard.

The flavonoid content per sample was determined using an aluminum chloride-based assay (Chang et al., 2002). 100 μL methanol extract and 200 μL water were combined with 300 μL NaNO_2_ (6 g/L) in a microcentrifuge tube. After 1 min of incubation at room temperature, 300 μL AlCl_3_⸱6H_2_O (22 g/L) was added. After 2 min of incubation at room temperature, 300 μL of NaOH (0.8 M) was added to the microcentrifuge tube. 200 μL of supernatant was transferred to a 96-well plate in triplicate, and the absorbance was read at 490 nm using the Synergy H1 Hybrid Multi-Mode Reader (BioTek). Absorbance values were converted to concentrations using catechin as a flavonoid compound standard.

### 2.5 PAL activity assays

Phenylalanine ammonia-lyase (PAL) activity was quantified in samples using a previously established protocol with several modifications (Sykłowska-Baranek et al., 2012). Fresh well-mixed culture samples in Safe-Lock microcentrifuge tubes (Eppendorf) were incubated at room temperature for several minutes, or until the cells settled to the bottom of the tube. Culture media was removed using a pipette tip and cells were washed twice with phosphate buffered saline (PBS) to remove any residual phenylalanine from the culture media. Following this, 1 mL assay buffer (50 mM Tris-HCl pH 8.0, 0.8 mM β-mercaptoethanol, 1% w/v polyvinylpyrrolidone) was added to each sample, along with the contents of one 2 mm BashingBead lysis tube (Zymo Research). Samples were homogenized in a bullet blender (Next Advance) at speed 8 for two 5 min cycles, then centrifuged at 20,000 ×g for 3 min. The following components were then transferred to a fresh microcentrifuge tube: 250 μL supernatant from cell lysate, 125 μL water, 250 μL Tris-HCl (pH 8.0), 125 μL L-phenylalanine (50 mM). Tubes were inverted several times to mix and incubated at room temperature for 1 h, following which 200 μL from each tube was transferred to a 96 well plate in triplicate and the absorbance was read at 290 nm. Enzyme activity was normalized to the cell line not treated with phenylalanine, PA, or CA.

### 2.6 Construction of binary vectors and transgenic Agrobacterium strains

All cloning was performed using NEB 10-beta competent *E. coli* (New England Biolabs) according to manufacturer’s instructions. Templates for construction of the no guide RNA control (ng) and PAL knockdown (PALg1) vectors were obtained from Addgene. Addgene #115488 was used as the no guide RNA control with no modifications. For generation of the PAL knockdown vector, the guide RNA sequence of Addgene #115486 was modified to 5′-CAC​AGG​CTT​AAG​CAC​CAC​CC-3′ using Gibson assembly. Guide RNA was designed in Benchling to target the 5′UTR of the *Taxus chinensis* PAL gene (Genbank accession #KF713533). Both ng and PALg1 constructs were then transformed into electrocompetent *Agrobacterium tumefaciens* EHA105 (GoldBio) according to manufacturer’s instructions.

### 2.7 Transformation and maintenance of transgenic *Taxus chinensis* cell lines

Transgenic *Taxus chinensis* 48.82A.3s cell lines were generated using a transformation protocol based on that described in Wilson et al. (2018). Glycerol stocks of *Agrobacterium tumefaciens* strains used for transformation were streaked on YEP plates (10 g/L yeast extract, 20 g/L peptone, 20 g/L agar) with 50 μg/mL kanamycin and rifampicin and grown for 2 days at 30°C, or until the formation of colonies. For each transformation, a single colony was used to inoculate 25 mL of YEP with 50 μg/mL kanamycin and rifampicin in a 125 mL flask. Flasks were incubated in a shaker-incubator at 30°C and 220 rpm until turbid, about 24–48 h. The OD_600_ of each culture was measured and cultures were pelleted by centrifugation at 4,000 ×g for 10 min. The supernatant was removed and cultures were resuspended to OD_600_ = 0.6 in 50 mL *Taxus* growth media (20 g/L sucrose, 0.5 mg/L naphthaleneacetic acid, 22.5 μg/L 6-benzylaminopurine, 3.21 g/L Gamborg B5 Basal Medium, pH 5.5) supplemented with a ×20 anti-necrotic solution (2.5 g/L ascorbic acid, 2.5 g/L citric acid, 14.6 g/L L-glutamine, 1 g/L L-cysteine) at 5% v/v. Acetosyringone (final concentration 200 µM) was then added to each culture and cultures were incubated at 30°C and 220 rpm for 30 min.

Healthy *Taxus chinensis* 48.82A.3s cultures on day 7–10 of a subculture cycle (mid-late log phase; approximately 15–20 mL packed cell volume per 50 mL culture) were sheared approximately 25 times with a 10 mL serological pipette to reduce aggregate size and improve infiltration. Cultures were then transferred to 50 mL conical tubes and allowed to settle without centrifugation for 10 min. Once there was a clear phase separation between cells and media, the supernatant was carefully removed using a serological pipette and replaced with the prepared *Agrobacterium* culture. The *Taxus-Agrobacterium* co-culture was then transferred back into a 125 mL flask and incubated at 23°C and 125 rpm for approximately 48 h. Following co-incubation, the co-culture was transferred into a 50 mL conical tube and cells were allowed to settle for 10 min, or until there was a clear phase separation between plant cells and *Agrobacterium* culture. *Agrobacterium* culture was removed using a serological pipette and replaced with an equal volume of *Taxus* growth media with anti-necrotic solution and 300 mg/L cefotaxime. Flasks were swirled approximately 10–20 times to mix and then poured into a conical tube to settle again. This media washing step was repeated 2 more times or until the supernatant appeared clear. The washed and resuspended *Taxus* culture was then transferred back into a 125 mL flask and incubated at 23°C and 125 rpm overnight to ensure complete eradication of *Agrobacterium.*

After overnight incubation, cultures were vacuum-filtered through a sterile Buchner funnel lined with Miracloth for approximately 30 s, or until biomass was lightly dried. The resulting biomass was then transferred to *Taxus* growth media plates (20 g/L sucrose, 0.5 mg/L naphthaleneacetic acid, 22.5 μg/L 6-benzylaminopurine, 3.21 g/L Gamborg B5 Basal Medium, 4 g/L Gelzan, pH 5.5) with anti-necrotic solution and 300 mg/L cefotaxime using a sterile spatula. Plates were wrapped with Parafilm and allowed to incubate at 23°C in the dark until friable callus growth was observed; approximately 4–6 weeks. Approximately 3 g of callus was then transferred into 125 mL flasks containing 50 mL of *Taxus* growth media with anti-necrotic solution, 300 mg/L cefotaxime, and 10 μg/mL hygromycin (selectable marker). Transgenic cultures were sheared approximately 50 times to break up cellular aggregates, then allowed to incubate at 23°C and 125 rpm for 21 days before taking culture samples for taxanes, DNA methylation, and gene expression. Samples for taxanes were evaporated overnight in an evaporative centrifuge as described in Section 2.2 and then analyzed for taxane content as described in Section 2.3. Samples for DNA and PAL gene expression were processed and stored as described for the gene expression samples in Section 2.2.

### 2.8 Estimation of PAL DNA methylation via MSRE-qPCR

To determine the approximate percentage of DNA methylation of the region targeted by the guide RNA, we used methylation-sensitive restriction enzyme qPCR (MSRE-qPCR). Genomic DNA from ng and PALg1 cell lines was extracted from frozen cell samples using the Wizard High MW DNA Extraction Kit (Promega) following the procedure for isolation from plant tissue. Instead of homogenization with liquid nitrogen, homogenization was performed using a Bullet Blender as described in Section 2.6, following which the procedure was performed according to manufacturer’s specifications. 100 ng of genomic DNA was then digested with the restriction enzyme McrBC (New England BioLabs) at 37°C for 4 h. Undigested controls were incubated with the same volume of nuclease-free water instead of restriction enzyme under the same conditions.

The digested genomic DNA and undigested controls were then amplified using qPCR. PrimeTime qPCR Probe Assays (Integrated DNA Technologies) were designed using IDT’s PrimerQuest design tool to target the same region as the guide RNA and contain at least 2 CpG sites (Supplementary Table S1). qPCR was then performed on an Applied Biosystems 7500 Real-Time PCR System (Thermo Fisher) using IDT’s PrimeTime 2x Gene Expression Master Mix. The difference in C_t_ values between the digested DNA and undigested control for each sample was used to determine the approximate percentage of DNA methylation of the targeted region. Due to the lack of a fully methylated control, a no template control was used instead to simulate fully methylated and thus completely digested DNA.

### 2.9 Quantification of PAL gene expression

For determination of PAL differential gene expression for both the inhibitor experiments (Section 2.2) and the experiments with transgenic cell lines (Section 2.7), total RNA was extracted using a Quick-RNA Plant Kit (Zymo Research). Cell samples were homogenized using a Bullet Blender as previously described in Section 2.5, following which RNA extraction was performed according to manufacturer’s instructions. Immediately following RNA extraction, cDNA was synthesized using approximately 1 μg of total RNA using the ProtoScript II First Strand cDNA Synthesis Kit (New England Biolabs). Expression of PAL was then quantified using an Applied Biosystems 7500 Real-Time PCR System (Thermo Fisher). PrimeTime qPCR Probe Assays (Integrated DNA Technologies) were designed for the PAL gene as well as the housekeeping gene (actin) using the IDT PrimerQuest design tool (Supplementary Table S1). HEX/ZEN/Iowa Black FQ probes were used for the housekeeping gene and FAM/ZEN/Iowa Black FQ probes were used for PAL, to enable in-well multiplexing. The log_2_fold change in expression of PAL relative to the respective control for each experiment was calculated using the double delta C_t_ method.

## 3 Results

### 3.1 Disruption of phenylpropanoid biosynthesis with chemical inhibitors affects PAL expression and activity

To begin to investigate the effects of phenylpropanoid pathway inhibition on taxane biosynthesis, we first turned to chemical inhibition of the pathway. Here, *Taxus chinensis* suspension cultures were treated with 100 µM piperonylic acid (PA) to inhibit C4H, 100 µM caffeic acid (CA) to inhibit 4CL, or 100 µM of both compounds. Notably, the genes coding for these enzymes have not yet been identified in *Taxus* species, necessitating the use of chemical inhibition rather than genetic manipulation. These inhibitors were tested with or without the presence of 1 mM exogenous phenylalanine to evaluate the effect of an increased precursor pool on flux to either phenylpropanoid or taxane metabolism.

**FIGURE 1:**
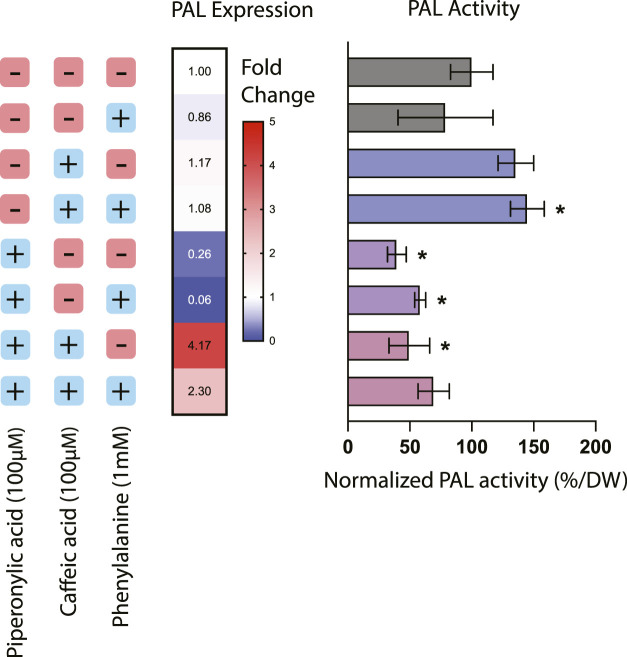
Phenylalanine ammonia-lyase (PAL) expression and activity in *Taxus chinensis* cell lines treated with phenylpropanoid pathway inhibitors piperonylic acid (PA) and caffeic acid (CA). PAL expression was measured using RT-qPCR. PAL activity was assessed using an enzyme activity assay and normalized by biomass dry weight. All values are normalized relative to the cell line not treated with PA or CA and without the addition of exogenous phenylalanine. Data represent biological triplicates ± standard deviation; *p < 0.05.

Treatment with phenylpropanoid pathway inhibitors had a significant effect on both expression and activity of PAL, the first committed step in the phenylpropanoid biosynthesis pathway from the precursor phenylalanine (Figure 1). The addition of PA alone resulted in a 60.5% decrease in PAL activity as well as a corresponding decrease in PAL expression of around 76%, suggesting that inhibition of C4H also affects regulation of this entry point into the pathway. This result is consistent with previous work in *Nicotiana tabacum* where silencing of C4H resulted in a decrease in both PAL activity and levels of downstream phenylpropanoid compounds, likely through product inhibition from cinnamic acid accumulation (Blount et al., 2000).

For the cell lines treated with CA or both CA and PA, effects of these inhibitors on PAL activity were decoupled from their effects on PAL expression. Treatment with CA alone resulted in an increase in PAL activity of around 35.6% but no significant effect on PAL expression, while treatment with both PA and CA together resulted in a 50.4% decrease in PAL activity but around a 4-fold increase in PAL expression. This discrepancy suggests that in these instances, transcription-level regulation is not necessarily coupled with protein-level regulation. For instance, in the case of treatment with both CA and PA, while PAL activity is decreased due to the putative action of C4H reducing PAL activity through product inhibition, it is possible that gene expression of PAL is actually increasing due to transcription-level regulatory mechanisms seeking to compensate for the decreased conversion of phenylalanine. One further consideration is also the fact that many plants have multiple copies of the PAL gene, ranging from 4 copies in the model plant *Arapidopsis thaliana* to 26 copies in the polyphenol-rich tomato (Liu et al., 2023). While PAL activity would be detectable from all possible PAL isoforms, it is likely that expression of multiple possible isoforms would not all be quantifiable via qPCR without designing multiple different probes. Thus, while to date only one copy of the PAL gene has been identified in *Taxus chinensis* (Genbank accession #KF713533), it is reasonable to assume that there may likely be other copies that have not yet been characterized that may be subject to different regulation than the specific copy of PAL targeted by these qPCR probes. More detailed mining for PAL isoforms in the *Taxus chinensis* genome would be needed to determine the number of copies of PAL and elucidate their regulation.

### 3.2 Treatment with piperonylic acid decreases biosynthesis of total flavonoids and phenolics

**FIGURE 2:**
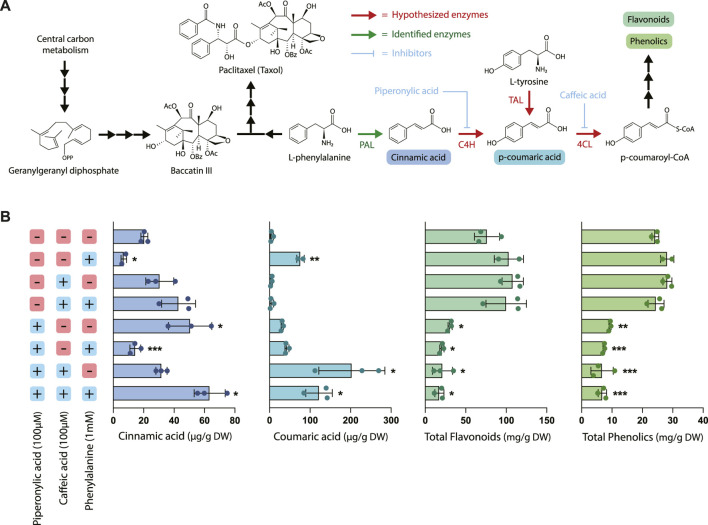
Effect of piperonylic acid (PA), caffeic acid (CA) and phenylalanine on phenylpropanoid pathway intermediates, flavonoids, and phenolics. **(A)** Pathway diagram illustrating the interactions between phenylpropanoid biosynthesis and taxane biosynthesis. Abbreviations: (PAL) phenylalanine ammonia-lyase, (C4H) cinnamate 4-hydroxylase, (TAL) tyrosine ammonia-lyase, (4CL) 4-coumarate-CoA ligase. **(B)** Concentrations of cinnamic acid, coumaric acid, total flavonoids, and total phenolics in *Taxus chinensis* cell cultures treated with 100 µM piperonylic acid, 100 µM caffeic acid, and/or 100 mM phenylalanine normalized by biomass dry weight. Statistical significance is relative to untreated control (top row); *p < 0.05, **p < 0.01, ***p < 0.001.

Next, to evaluate the effects of chemical inhibition on phenylpropanoid pathway products, the two early-stage phenylpropanoid precursors cinnamic acid and coumaric acid were quantified via UPLC and total phenolics and flavonoids were estimated using colorimetric assays (Figure 2). The addition of exogenous phenylalanine without the presence of inhibitors resulted in both a decrease in cinnamic acid accumulation and an increase in coumaric acid accumulation, indicating that under conditions of excess phenylalanine bioavailability, C4H is more active in converting cinnamic acid to coumaric acid. Since under these circumstances PAL activity was slightly decreased (78.8% that of the control) and PAL expression is largely unchanged, this suggests that the accumulation of coumaric acid is likely primarily due to regulation at C4H rather than PAL. Further, treatment with PA alone resulted in a significantly higher accumulation of cinnamic acid as expected due to inhibition of C4H, but also a slightly elevated level of coumaric acid. One potential explanation for this phenomenon is that through initial inhibition of coumaric acid formation via inhibition of C4H, the enzyme TAL (which often works in tandem with PAL) became more active in converting tyrosine to coumaric acid due to the lack of production of coumaric acid by 4CL.

Cultures treated with CA alone did not accumulate significantly higher levels of coumaric acid, which was unexpected due to the proposed mechanism of caffeic acid inhibiting conversion of coumaric acid to coumaroyl-CoA via 4CL. It is possible that this concentration of caffeic acid alone was insufficient to inhibit 4CL via product inhibition for the entire length of the experiment because caffeic acid is consumed as a substrate in the reaction. In contrast, the same concentration of PA provided sufficient inhibition because it is hypothesized to irreversibly bind to its substrate and is known to be active in concentrations as low at 10 µM (Nkomo et al., 2022). However, with both CA and PA treatment we observed a dramatic increase in coumaric acid accumulation as well as a smaller increase in cinnamic acid accumulation, despite the decreased PAL activity observed for these treatments. This indicates that CA is in fact inhibiting coumaric acid conversion, at least when combined with treatment with PA, and suggests that there may be some emergent effects on the pathway from treatment with both inhibitors simultaneously.

While treatment with phenylalanine, CA, and PA resulted in complex and sometimes unexpected effects on PAL activity and accumulation of the earlier pathway intermediates, the effect on accumulation of total phenolics and flavonoids was quite straightforward. All treatments with PA resulted in a statistically significant decrease in both total flavonoids and total phenolics (approximately a 2- to 5-fold decrease, depending on the treatment), while phenylalanine feeding alone or treatment with CA had no effect on accumulation of either of these metabolite classes. This decrease in metabolite accumulation for the cell lines treated with PA is consistent with the decreases in PAL activity observed in Figure 1 and previously characterized effects of PA on downstream phenylpropanoid metabolite accumulation (Nkomo et al., 2022). Interestingly, while treatment with PA and CA resulted in dramatic increases in coumaric acid accumulation, there was still an overall decrease in the total accumulation of both phenolics and flavonoids despite elevated levels of these early pathway intermediates. This suggests that there are likely additional regulatory mechanisms downstream of coumaric acid that are affected by the presence of these inhibitors, illustrating the complexity and level of cross-talk in regulation of this pathway.

### 3.3 Inhibition of phenylpropanoid metabolism with chemical inhibitors increases production of paclitaxel and its precursors

**FIGURE 3:**
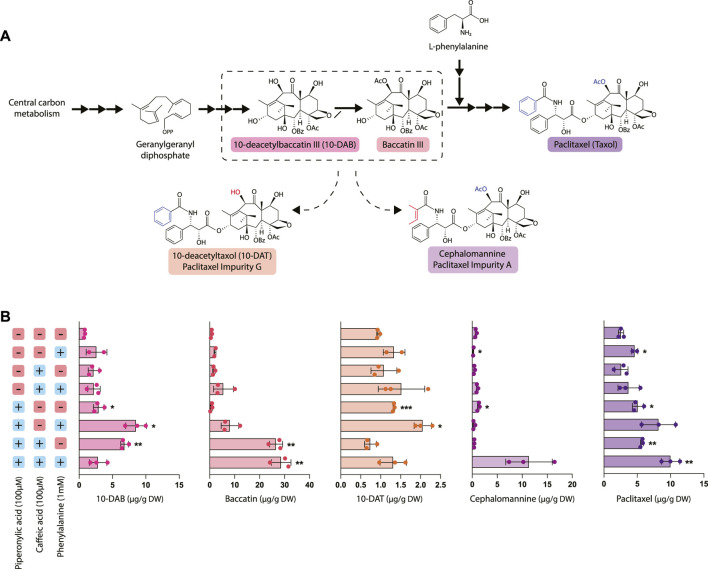
Effect of piperonylic acid (PA), caffeic acid (CA) and phenylalanine on taxane biosynthesis. **(A)** Simplified pathway depicting biosynthesis of paclitaxel from the precursor geranylgeranyl diphosphate. 10-deacetyltaxol (10-DAT) and cephalomannine are taxane derivatives synthesized from currently unknown pathways likely derived from paclitaxel biosynthetic pathway intermediates **(B)** Concentrations of 10-DAB, baccatin III, 10-DAT, cephalomannine, and paclitaxel in *Taxus chinensis* cell cultures treated with 100 µM piperonylic acid, 100 µM caffeic acid, and/or 100 mM normalized by biomass dry weight. Statistical significance is relative to untreated control (top row); *p < 0.05, **p < 0.01, ***p < 0.001.

To elucidate the relationship between taxane and phenylpropanoid biosynthesis, we evaluated the effect of phenylalanine precursor feeding and phenylpropanoid pathway inhibition with CA and PA on taxane accumulation (Figure 3). In particular, we measured the accumulation of five different taxanes: 10-deacetylbaccatin III (10-DAB), baccatin III, and paclitaxel, which occur in the canonical paclitaxel biosynthesis pathway, as well as the two taxane side products 10-deacetyltaxol (10-DAT) and cephalomannine. These taxanes are two of dozens of “paclitaxel impurities,” which are compounds closely related to paclitaxel whose biosynthetic pathways are not yet characterized. While not much is currently known about biosynthesis of these compounds, they are possibly derived from taxane pathway intermediates like 10-DAB and baccatin III (Croteau et al., 2006; Li et al., 2017). Through studying their accumulation patterns, we aim to gain insight into their biosynthetic pathways and pathway regulation.

Most notably, treatment with PA significantly increased paclitaxel accumulation, with the largest increase (approximately a 3.5-fold increase over the control) observed in cultures treated with all three compounds (phenylalanine, PA, and CA). This increase in paclitaxel accumulation was strongly inversely correlated with the decreased PAL activity and phenolic/flavonoid accumulation observed in the cell lines treated with PA and interestingly, not PAL expression. This strongly suggests that repression of phenylpropanoid biosynthesis through decreased activity of PAL and/or C4H reroutes phenylalanine toward synthesis of other natural products, such as taxanes. 10-DAT production appeared to be roughly correlated with paclitaxel production, with the exception of the cultures treated with all three compounds that instead produced a large amount of cephalomannine. Additionally, 10-DAT production was consistently slightly increased with the addition of phenylalanine, while cephalomannine production did not follow this same trend. This suggests that perhaps 10-DAT is synthesized from either paclitaxel itself or another late-stage intermediate that occurs after the addition of the phenylalanine side chain to baccatin III, since production of 10-DAT and paclitaxel is possibly co-regulated. Conversely, since cephalomannine is not affected by the addition of phenylalanine in the same way that 10-DAT and paclitaxel are, it may be synthesized from a different, earlier precursor.

Unexpectedly, inhibition of phenylpropanoid metabolism not only affected paclitaxel accumulation, but also the accumulation of other taxanes upstream of the point in the pathway where phenylalanine attaches to form the side chain of paclitaxel (after the synthesis of baccatin III). Treatment with PA generally resulted in a significant increase in 10-DAB accumulation with the exception of the cell line treated with all three compounds (phenylalanine, CA, and PA). This line had significantly increased accumulation of both paclitaxel and cephalomannine, suggesting that rather than accumulating high levels of 10-DAB, it was instead converted to these downstream products. Further, cell lines treated with both CA and PA had profoundly increased levels of baccatin III accumulation—nearly 30 times higher than that of the control. These data suggest that while treatment with CA alone was insufficient to affect flux to taxane biosynthesis, the emergent effect of PA and CA together is more effective at directing flux than PA alone. Additionally, this points to DBAT, the gene controlling conversion of 10-DAB to baccatin, as an important gene involved in controlling taxane biosynthesis. This is consistent with previous data establishing DBAT as a putative rate-controlling step that is known to be subject to complex transcriptional regulation (Zhang et al., 2011; Li et al., 2013; Chen et al., 2022).

### 3.4 Repression of PAL by CRISPR-mediated DNA methylation specifically upregulates paclitaxel biosynthesis

While chemical inhibition of metabolism is advantageous for certain circumstances, we also wanted to pioneer novel approaches for targeted genetic manipulation of metabolism. In particular, while transient overexpression and knockdown of genes has been used previously in *Taxus* cell culture for gene characterization (Zhang et al., 2015; Zhang et al., 2018; Chen et al., 2021), we wanted to study the effects of repressing phenylpropanoid biosynthesis in a stably transformed cell line representative of what might be used in an industrial process (Wilson et al., 2018). There have been some efforts to increase paclitaxel titers in *Taxus* plant cell culture through pathway overexpression (Zhang et al., 2011), but CRISPR-based repression of competing pathways has not yet been explored to our knowledge. To this end, we chose to knock down the PAL gene using a CRISPR-based approach, since this was the only gene in the phenylpropanoid biosynthetic pathway that had specifically been characterized in *Taxus chinensis*. Due to the importance of the PAL gene in producing natural products vital to plant cell health (such as lignin, which is vital for formation of the plant cell wall), we chose to use targeted DNA methylation as an approach to modestly decrease, but not completely knock down expression of this gene.

As a template, we chose to use a dCas9-SunTag system for engineering DNA methylation in the model plant *Arabidopsis thaliana* developed by the Jacobsen lab (Papikian et al., 2019). This work adapted the dCas9-SunTag system for use in engineering DNA methylation in plants by using the catalytic domain of a DNA methyltransferase from *Nicotiana tabacum* (NtDRMcd) as well as plant-specific promoters and terminators, which resulted in *Arabidopsis* plants that had heritable and stable epialleles. The original work found that in general, guide RNAs placed near the transcription start site of the targeted gene tended to be more effective, so we designed our guide RNA to bind to the 5′ untranslated region (5′UTR) of the PAL gene, with the 3′ end of the guide RNA placed 6 base pairs upstream of the transcription start site. We hypothesized that targeting the gRNA to the 5′UTR would broadly methylate both the 5′UTR and the unidentified promoter region upstream, since in the original manuscript the authors found that the methyltransferase was able to methylate a large region of DNA spanning up to approximately 2 kb around the guide RNA. Finally, the absence of a sequenced *Taxus chinensis* genome at the time of these experiments also necessitated the use of a no guide RNA control, since we did not want to design a scrambled guide RNA control that had unpredictable targeting activity.

**FIGURE 4:**
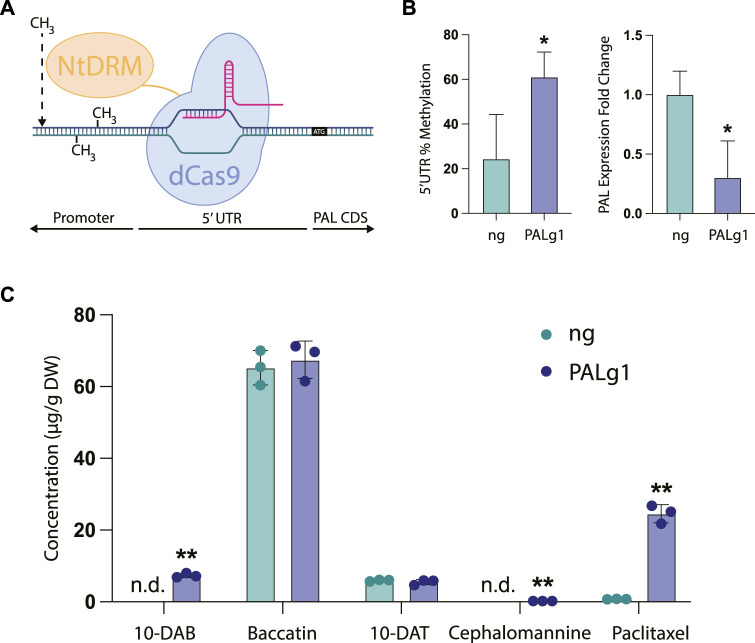
CRISPR/dCas9-NtDRMcd targeted methylation of PAL in *Taxus chinensis*. **(A)** Schematic of CRISPR/dCas9-NtDRMcd mechanism of action. Guide RNA was designed to allow dCas9 to bind to the 5′ untranslated region (5′UTR) of the PAL gene to enable targeted DNA methylation by the NtDRMcd domain. **(B)** Approximate DNA methylation of the PAL 5′UTR measured by MSRE-qPCR and PAL gene expression measured by RT-qPCR for the no guide RNA control (ng) and PAL methylated (PALg1) cell lines. **(C)** Concentrations of 10-DAB, baccatin III, 10-DAT, cephalomannine, and paclitaxel in engineered ng and PALg1 cell lines normalized by biomass dry weight. Statistical significance represents difference between ng and PALg1 cell lines; *p < 0.05, **p < 0.01.

In our stably transformed cell lines, we observed a significant increase in DNA methylation of the region targeted by the guide RNA from approximately 24% in the no guide RNA control to around 61% in the PAL knockdown line PALg1 (Figure 4B). This was coupled with a significant decrease in PAL gene expression, with expression in the PALg1 line approximately 30% that of the ng control. These results confirm that our guide RNA is targeting the desired region, and that this increased DNA methylation is resulting in changes in gene expression. While we did not specifically evaluate DNA methylation of the PAL promoter region since it has not yet been characterized, from these data we can assume that methylation of the 5′UTR was sufficient to cause gene repression and/or methylation of the promoter region was similarly increased as expected from the original study.

As expected, we also observed differential accumulation of taxanes in the PALg1 and ng *Taxus chinensis* cell lines (Figure 4C). While with the chemical inhibitors (Figure 3B) we observed approximately a 3.5-fold increase in paclitaxel accumulation for the cell line treated with all three compounds (phenylalanine, PA, and CA) relative to the control, here we saw approximately a 25-fold increase in paclitaxel accumulation from 0.76 μg/g DW in the ng cell line to 24.5 μg/g DW in the PALg1 cell line. Additionally, 10-DAB and cephalomannine were not detected in the ng line but detected in small amounts in the PALg1 line, while there was no difference in 10-DAT or baccatin production between the two lines. This suggests that by directly inhibiting PAL, we can achieve a more specific increase in yield of paclitaxel when compared to chemically inhibiting downstream steps in the pathway, which resulted in a more significant increase in the yield of other taxane intermediates and side products.

## 4 Discussion

Plant cell culture systems are becoming increasingly popular for large-scale production of pharmaceuticals, supplements, and other complex plant natural products of social interest. However, a major challenge with improving yield of products of interest in plant cell culture is the number of complex and often intertwined metabolic pathways, each subject to their own intricate pathway regulation. In the case of *Taxus* species, the enzyme PAL acts as a bridge between two competing pathways: phenylpropanoid metabolism and taxane metabolism, which both utilize phenylalanine as a precursor. Often in the context of metabolic engineering we solely consider manipulating the pathway of interest, but here, we show that manipulating cooperating or competing pathways can be of equal or even greater importance in plant cell systems. In this work, we demonstrated that we can achieve up to a 25-fold increase in paclitaxel accumulation by downregulating phenylpropanoid metabolism at multiple different steps in the pathway. Additionally, we uncovered several interesting insights about phenylpropanoid biosynthesis in *Taxus* species and its relationship with taxane biosynthesis.

First, we found that treatment with piperonylic acid, an irreversible chemical inhibitor of C4H (the second step in phenylpropanoid biosynthesis after PAL) resulted in both a decrease in accumulation of total phenolics and flavonoids and an increase in paclitaxel biosynthesis. This was also correlated with a decrease in PAL activity, indicating that phenylpropanoid biosynthesis and taxane biosynthesis likely compete for metabolic flux despite the fact that they are often co-regulated. While the effect of piperonylic acid on phenolic and flavonoid biosynthesis did not change significantly when CA or phenylalanine was added, there was a clear increase in paclitaxel accumulation when all three compounds were used in tandem. This indicates that while inhibition of C4H using PA plays an important regulatory role in controlling synthesis of phenolics and flavonoids, there are likely other uncharacterized effects on pathway regulation that are more important for paclitaxel production.

The fact that manipulating phenylpropanoid metabolism also affected metabolism of early taxane intermediates can potentially be explained by considering the interplay of phenylpropanoid metabolism with the plant stress response. In nature, production of secondary metabolites such as paclitaxel is often dynamically upregulated in response to stress, and the effect of elicitors on induction of taxane biosynthesis in *Taxus* plant cell culture is an extremely well-studied phenomenon (Yukimune et al., 1996; Zhang et al., 2000; Li et al., 2012; Lenka et al., 2015; Zhang et al., 2015). In particular, it is likely that treatment with PA could increase production of salicylic acid and reactive oxygen species, inducing the stress response and thus upregulating production of taxanes. Salicylic acid is a plant signaling hormone also commonly used as an eliciting agent that is generally accepted to be synthesized through two possible pathways in plants: through the enzyme isochorismate synthase (ICS) or from cinnamic acid synthesized by PAL (Lefevere et al., 2020; Liu et al., 2022). Since treatment with PA increased levels of cinnamic acid in our cell lines (Figure 2B), it is possible that this also upregulated biosynthesis of salicylic acid, which is known to be a positive regulator of taxane biosynthesis (Wang et al., 2007; Sarmadi et al., 2018). This would be consistent with previous work that found that treatment with PA increased markers associated with oxidative stress and oxygen scavenging enzyme activity in chia seedlings, prompting increased production of signaling hormones like SA that are involved in protection from oxidative damage (Nkomo et al., 2022).

In addition, at least one transcription factor has been identified that upregulates taxane biosynthesis specifically in response to salicylic acid (Chen et al., 2021). This salicylic acid-responsive transcription factor was found to bind specifically to the DBAT promoter, resulting in a large increase in DBAT expression. When transiently overexpressed, this transcription factor induced activation of most taxane biosynthetic pathway genes, indicating that in addition to directly upregulating DBAT, it likely upregulates these other pathway genes through indirect mechanisms. This general upregulation of taxane biosynthetic pathway genes would explain the effects of PA on increasing not only paclitaxel production, but also the production of the precursors 10-DAB and baccatin.

Second, while PA and CA are inhibitors of the second and third steps in phenylpropanoid biosynthesis (C4H and 4CL, respectively), they also had off-target effects on both PAL activity and expression. This indicates that at least these initial steps in the pathway are subject to complex transcriptional regulation and likely form a tight regulatory unit, as has been reported in other plants (Koopmann et al., 1999; Zhang et al., 2020). There was also a more complex relationship than expected between PAL activity and expression, suggesting distinct regulation at the transcriptional level and the protein level that will require more in-depth study to fully deconvolute. Interestingly, we also found that while treatment with PA resulted in variable accumulation of cinnamic acid and coumaric acid when combined with other treatments, there was a very consistent decrease in total flavonoid and phenolic accumulation in these lines. This suggests that perhaps there are some additional downstream effects of C4H inhibition not captured within this study that may be responsible for downregulating production of these end products. In future work, it would be useful to identify the effects of C4H enzyme inhibition on additional phenylpropanoid biosynthetic pathway intermediates occurring later in the pathway in order to identify the mechanism of this clear decrease in total phenolics and flavonoids.

We also demonstrated that repression of PAL using a CRISPR/dCas9-based tool was highly effective at specifically increasing flux to paclitaxel production rather than precursors or side products, illustrating the efficacy of targeted engineering of competing pathways for products of interest. Interestingly, the patterns of metabolite accumulation in these transgenic cell lines (Figure 4) were notably different than those observed with the non-transgenic cell lines in Figure 3, where there was comparatively more 10-DAB and less baccatin in all cell lines. This can potentially be explained by two different mechanisms.

First, the differences in metabolite accumulation between the lines treated with chemical inhibitors and the transgenic cell lines may be a byproduct of transformation. *Agrobacterium*-based transformation has been shown to result in changes to the transcriptome in several plant species and in particular, upregulation of genes associated with hormone signaling and stress (Duan et al., 2017; Wang et al., 2023). This could contribute to the differential taxane accumulation patterns in transformed *Taxus* cell cultures compared to untransformed ones. Furthermore, *Taxus* cell cultures are known to produce phenylpropanoid pathway derivatives in response to stress, so if there is a higher amount of flux through the phenylpropanoid biosynthetic pathway due to the stress associated with transformation, this could explain the large increase in paclitaxel observed in the PALg1 cell line since there is more metabolic flux available to be rerouted to paclitaxel production.

Secondly, in the manuscript we adapted this tool from, the authors noted that there was a slightly increased level of global DNA methylation throughout the genome in their transgenic plants due to non-specific methylation activity of NtDRM (Papikian et al., 2019). This could potentially explain the lower levels of paclitaxel accumulation in the ng cell line compared to untransformed Taxus cell lines (Figure 3C) if some of the later-stage genes in the paclitaxel biosynthetic pathway are non-specifically methylated. It is also important to note that a BLAST search of the guide RNA against the *Taxus chinensis* genome confirmed that this guide RNA had a single target, so it is unlikely that these differences are due to off-target activity of the guide RNA. However, it is difficult to discern which effects are due to hypermethylation and which are simply due to the stress and other metabolic changes associated with transformation; further genomics and transcriptomic work would be needed to deconvolute these effects.

Additionally, despite the fact that many plants have multiple copies of the PAL gene, we found here that methylating presumably only a single copy of the gene was sufficient to drastically increase paclitaxel production by over 25-fold. This could indicate that *Taxus* species only have a single copy of the PAL gene, but that would be highly unusual among plant species, especially for a plant that produces many natural products dependent on phenylalanine as a precursor. At present, the lack of knowledge of the number of copies of PAL in the *Taxus* genome limits in-depth interpretation of these results. As genomic and transcriptomic resources for non-model plants such as *Taxus* species become more abundant, conclusively determining the number of copies of the PAL gene in the genome and multiplexing guide RNAs to target multiple different copies could help further inform how downregulation of PAL affects taxane biosynthesis. This could also be used to more specifically tune the degree of knockdown of PAL to prevent potential deleterious effects on cell wall architecture, due to the importance of PAL in controlling flux to lignin biosynthesis.

More broadly, this work provides a proof-of-concept for the use of epigenetic engineering strategies to manipulate metabolism in plant cell culture and opens the door for more in-depth studies of secondary metabolism. In this work, we revealed some of the intricacies in regulation of the phenylpropanoid biosynthetic pathway, but there are likely many more complex interactions that have yet to be discovered. Using transcriptomics to study *Taxus* cell lines where the phenylpropanoid pathway was inhibited using both genetic and chemical methods could help further elucidate the coregulation of taxane and phenylpropanoid biosynthesis. Additionally, by identifying genes that are coregulated with PAL, we could likely identify more genes involved in the phenylpropanoid biosynthetic pathway in *Taxus* species, enabling further genetic manipulation of the pathway. These transcriptomic studies could be combined with enzyme activity assays validated for different phenylpropanoid pathway enzymes (such as TAL) to understand the contributions of regulation on both the transcriptional and protein levels.

Another natural extension of this work would be to study the efficacy of gene activation through targeted demethylation of genes that are proposed to be downregulated through increased DNA methylation, such as BAPT (Sanchez-Muñoz et al., 2018). This tool could potentially be used to recover secondary metabolite production in continuously subcultured plant cell lines and thus improve their useful lifetime and productivity. However, applying this more broadly in plant cell culture systems would require more extensive epigenomics work in order to identify loci that are both known to be methylated and play important roles in controlling plant metabolism. Ultimately, we envision using these tools as a complement to other CRISPR-based technologies (such as CRISPRi or CRISPRa) and traditional overexpression-based pathway engineering approaches. Notably, this targeted DNA methylation tool required minimal adaptation from its original intended chassis (*Arapidopsis thaliana*) to a non-model plant (*Taxus chinensis*), likely due to the broad host suitability of the promoters used. This shows that other similar tools developed for model plants may be easily transferrable to non-model plants such as *Taxus chinensis*, which could rapidly speed the development of more modern engineering strategies for these chassis.

In conclusion, this work uniquely investigated the effect of manipulating phenylpropanoid metabolism on taxane biosynthesis in *Taxus* plant cell culture using both chemical inhibition and CRISPR-based gene repression. To optimize production of compounds of interest in organisms like plants that produce such a diverse array of natural products, our results demonstrate that it is imperative to manipulate not only the pathway of interest, but also competing or cooperating secondary metabolic pathways. By combining repression of side pathways using the tools developed here with activation of desired pathways through metabolic engineering, we could likely achieve unprecedented levels of control over flux through plant secondary metabolism. Through the application of these novel tools for manipulating metabolism, we have the potential to rationally optimize medicinal plant cell systems as next-generation chassis for the production of societally valuable compounds.

## Scope statement

Plant cell culture biomanufacturing is rapidly becoming an effective strategy for production of high-value plant natural products, such as chemotherapeutics (paclitaxel), vaccine adjuvants (QS-21), and nutraceuticals (cacao, coffee, echinacea). Despite the rapid growth of these technologies, strategies for metabolic engineering of plant cell cultures remain underdeveloped and hyper-focused on engineering the primary metabolic pathway of interest. Here, we show that manipulating phenylpropanoid biosynthesis in *Taxus* species using CRISPR-guided DNA methylation is highly effective at increasing taxane production—increasing paclitaxel accumulation by over 25-fold. While CRISPR-based technologies have been used to engineer well-studied plants, here we describe one of the first applications of CRISPR to a non-model plant cell culture system. Furthermore, this work illustrates that manipulating DNA methylation of key secondary metabolic genes is a useful tool to consider for repression of metabolism along with other more established technologies such as CRISPRi. This demonstrates the power and potential of these tools to manipulate metabolism in plant cell systems for metabolic engineering applications and paves the way for further rational cell line engineering in this rapidly growing field.

## Data Availability

The original contributions presented in the study are included in the article/Supplementary Material, further inquiries can be directed to the corresponding author.
