# Supplementary material for: Targeted control of supporting pathways in paclitaxel biosynthesis with CRISPR-guided methylation
Source: Front Bioeng Biotechnol. 2023 Oct 17;11:1272811. doi: 10.3389/fbioe.2023.1272811 (PMC10616794; doi:10.3389/fbioe.2023.1272811)
Supplement: Supplementary file 1 [file DataSheet1.docx]

Supplementary Material

**Targeted control of supporting pathways in paclitaxel biosynthesis with CRISPR-guided methylation**

Cassandra Brzycki Newton^1^, Eric M. Young^1^, Susan C. Roberts^1^

^1^Department of Chemical Engineering, Worcester Polytechnic Institute, Worcester, MA 01609

Correspondence: Susan Roberts, [scroberts@wpi.edu](mailto:scroberts@wpi.edu)

# Supplementary Figures and Tables

## Supplementary Figures


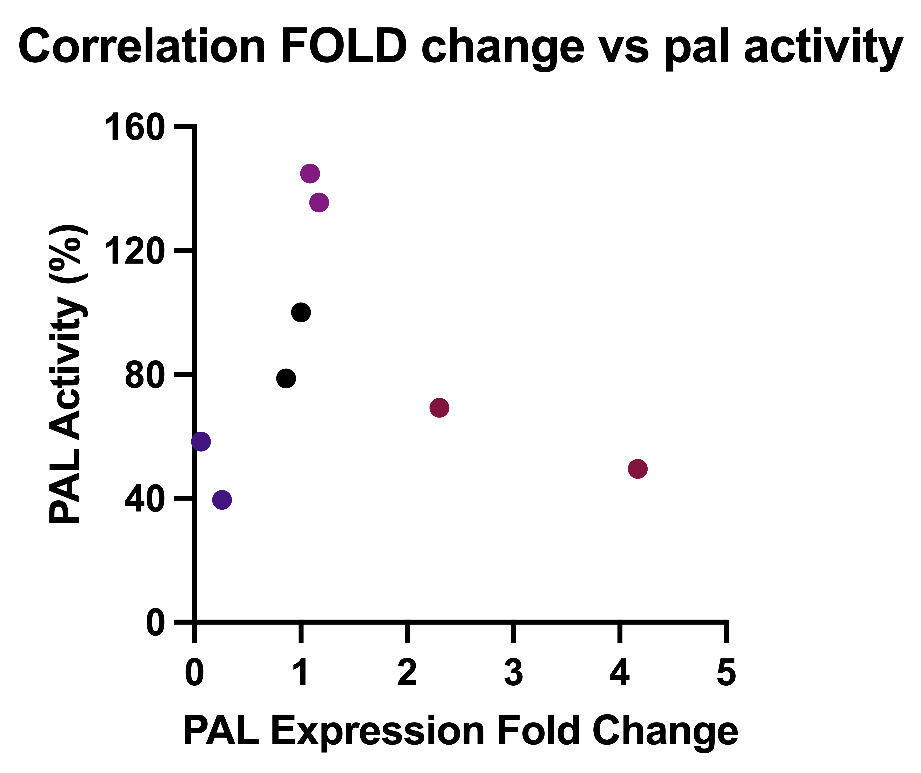


**Supplemental Figure 1. Correlation between phenylalanine ammonia-lyase (PAL) expression and activity in Taxus chinensis cell lines treated with phenylpropanoid pathway inhibitors piperonylic acid (PA) and caffeic acid (CA).** PAL expression was measured using RT-qPCR. PAL activity was assessed using an enzyme activity assay and normalized by biomass dry weight. All values are normalized relative to the cell line not treated with PA or CA and without the addition of exogenous phenylalanine. Data represent biological triplicates +/- standard deviation.

## Supplementary Tables

**Supplemental Table 1. qPCR probes and primers for quantification of gene expression and DNA methylation.**

| **Gene** | **Type** | **Oligo** | **Sequence** |
| --- | --- | --- | --- |
| Actin | Expression | F Primer | TCCGCGATGTGAAAGAGAAG |
|  |  | Probe | /5HEX/TAGCACTGG/ZEN/ACTTTGAGCAGGAGC/3IABkFQ/ |
|  |  | R Primer | TGTCCATCAGGAAGCTCATAAC |
| PAL | Expression | F Primer | CAGAAACCCAAGCAGGATAGA |
|  |  | Probe | /56-FAM/AGTGCGAGC/ZEN/GGCAACTAAGATGAT/3IABkFQ/ |
|  |  | R Primer | GTTGTCGTTCACCGAGTTTATTT |
| PAL | Methylation | F Primer | CGTTGACGCACAGGCTTA |
|  |  | Probe | /56-FAM/CCGCCGCGA/ZEN/TCATGGAATACATTCT/3IABkFQ/ |
|  |  | R Primer | TAGCAGCCGCTTTCATGTAG |
